# Supplementary material for: Resolving the full spectrum of human genome variation using Linked-Reads
Source: Genome Res. 2019 Apr;29(4):635–45. doi: 10.1101/gr.234443.118 (PMC6442396; doi:10.1101/gr.234443.118)
Supplement: Supplemental Material [file supp_gr.234443.118_Supplemental_longranger-2.2.zip › longranger-2.2.tar.bz2!/lr2.2-public/lib/stan/cmdstan/stan/doc/api/html/integrate__ode_8hpp_source.html]

Stan: src/stan/math/ode/integrate\_ode.hpp Source File


|  |  |
| --- | --- |
|  | Stan  2.5.0  probability, sampling & optimization |


- Main Page
- Related Pages
- Namespaces
- Classes
- Files

- File List
- File Members

All Classes Namespaces Files Functions Variables Typedefs Enumerations Enumerator Friends Macros Pages

- src
- stan
- math
- ode

integrate\_ode.hpp

Go to the documentation of this file.

1 #ifndef STAN\_\_MATH\_\_ODE\_\_INTEGRATE\_ODE\_HPP

2 #define STAN\_\_MATH\_\_ODE\_\_INTEGRATE\_ODE\_HPP

3

4 #include <ostream>

5 #include <vector>

6 #include <boost/numeric/odeint.hpp>

7 #include <stan/meta/traits.hpp>

8 #include <stan/math/functions/value\_of.hpp>

9 #include <stan/math/error\_handling/check\_less.hpp>

10 #include <stan/math/error\_handling/check\_finite.hpp>

11 #include <stan/math/error\_handling/matrix/check\_nonzero\_size.hpp>

12 #include <stan/math/error\_handling/matrix/check\_ordered.hpp>

13

14 #include <stan/math/ode/coupled\_ode\_system.hpp>

15 #include <stan/math/ode/coupled\_ode\_observer.hpp>

16

17 namespace stan {

18

19  namespace math {

20

59  template <typename F, typename T1, typename T2>

60  std::vector<std::vector<typename stan::return\_type<T1,T2>::type> >

61  integrate\_ode(const F& f,

62  const std::vector<T1> y0,

63  const double t0,

64  const std::vector<double>& ts,

65  const std::vector<T2>& theta,

66  const std::vector<double>& x,

67  const std::vector<int>& x\_int,

68  std::ostream\* msgs) {

69  using boost::numeric::odeint::integrate\_times;

70  using boost::numeric::odeint::make\_dense\_output;

71  using boost::numeric::odeint::runge\_kutta\_dopri5;

72

73  stan::math::check\_finite("integrate\_ode(%1%)", y0, "initial state",

74  static\_cast<double\*>(0));

75  stan::math::check\_finite("integrate\_ode(%1%)", t0, "initial time",

76  static\_cast<double\*>(0));

77  stan::math::check\_finite("integrate\_ode(%1%)", ts, "times",

78  static\_cast<double\*>(0));

79  stan::math::check\_finite("integrate\_ode(%1%)", theta, "parameter vector",

80  static\_cast<double\*>(0));

81  stan::math::check\_finite("integrate\_ode(%1%)", x, "continuous data",

82  static\_cast<double\*>(0));

83

84  stan::math::check\_nonzero\_size("integrate\_ode(%1%)", ts, "times",

85  static\_cast<double\*>(0));

86  stan::math::check\_nonzero\_size("integrate\_ode(%1%)", y0, "initial state",

87  static\_cast<double\*>(0));

88  stan::math::check\_ordered("integrate\_ode(%1%)", ts, "times",

89  static\_cast<double\*>(0));

90  stan::math::check\_less("integrate\_ode(%1%)",t0, ts[0], "initial time",

91  static\_cast<double\*>(0));

92

93  const double absolute\_tolerance = 1e-6;

94  const double relative\_tolerance = 1e-6;

95  const double step\_size = 0.1;

96

97  // creates basic or coupled system by template specializations

98  coupled\_ode\_system<F, T1, T2>

99  coupled\_system(f, y0, theta, x, x\_int, msgs);

100

101  // first time in the vector must be time of initial state

102  std::vector<double> ts\_vec(ts.size() + 1);

103  ts\_vec[0] = t0;

104  for (size\_t n = 0; n < ts.size(); n++)

105  ts\_vec[n+1] = ts[n];

106

107  std::vector<std::vector<double> > y\_coupled(ts\_vec.size());

108  coupled\_ode\_observer observer(y\_coupled);

109

110  // the coupled system creates the coupled initial state

111  std::vector<double> initial\_coupled\_state

112  = coupled\_system.initial\_state();

113

114  integrate\_times(make\_dense\_output(absolute\_tolerance,

115  relative\_tolerance,

116  runge\_kutta\_dopri5<std::vector<double>,

117  double,

118  std::vector<double>,

119  double>() ),

120  coupled\_system,

121  initial\_coupled\_state,

122  boost::begin(ts\_vec), boost::end(ts\_vec),

123  step\_size,

124  observer);

125

126  // remove the first state corresponding to the initial value

127  y\_coupled.erase(y\_coupled.begin());

128

129  // the coupled system also encapsulates the decoupling operation

130  return coupled\_system.decouple\_states(y\_coupled);

131  }

132

133  }

134

135 }

136

137 #endif

stan::math::check\_ordered

bool check\_ordered(const char \*function, const Eigen::Matrix< T\_y, Eigen::Dynamic, 1 > &y, const char \*name, T\_result \*result)

Return true if the specified vector is sorted into increasing order.

**Definition:** check\_ordered.hpp:29

coupled\_ode\_system.hpp

stan::math::check\_finite

bool check\_finite(const char \*function, const T\_y &y, const char \*name, T\_result \*result)

Checks if the variable y is finite.

**Definition:** check\_finite.hpp:52

stan::math::integrate\_ode

std::vector< std::vector< typename stan::return\_type< T1, T2 >::type > > integrate\_ode(const F &f, const std::vector< T1 > y0, const double t0, const std::vector< double > &ts, const std::vector< T2 > &theta, const std::vector< double > &x, const std::vector< int > &x\_int, std::ostream \*msgs)

Return the solutions for the specified system of ordinary differential equations given the specified ...

**Definition:** integrate\_ode.hpp:61

traits.hpp

coupled\_ode\_observer.hpp

check\_ordered.hpp

stan::math::coupled\_ode\_observer

Observer for the coupled states.

**Definition:** coupled\_ode\_observer.hpp:15

stan::math::e

double e()

Return the base of the natural logarithm.

**Definition:** constants.hpp:86

check\_finite.hpp

value\_of.hpp

stan::math::coupled\_ode\_system

Base template class for a coupled ordinary differential equation system, which adds sensitivities to ...

**Definition:** coupled\_ode\_system.hpp:27

stan::math::check\_less

bool check\_less(const char \*function, const T\_y &y, const T\_high &high, const char \*name, T\_result \*result)

**Definition:** check\_less.hpp:52

check\_nonzero\_size.hpp

check\_less.hpp

stan::math::check\_nonzero\_size

bool check\_nonzero\_size(const char \*function, const T\_y &y, const char \*name, T\_result \*result)

Return true if the specified matrix/vector is of non-zero size.

**Definition:** check\_nonzero\_size.hpp:33


---

[ Stan Home Page ]

*© 2011–2014,
Stan Development Team.*
